# Current Evidence on Gene-Based and Epigenetic Therapeutic, Diagnostic, and Prognostic Strategies in Epithelial-Derived Carcinomas: A Scoping Review

**DOI:** 10.3390/biomedicines14102234

**Published:** 2026-10-02

**Authors:** Joshua George Thomas, Mohammed Ayyub, Lovely M. Annamma, Prathibha Prasad, Sabrin Ali Azim, Sudhir Varma, Biji Thomas George, Al Moutassem Billah Khair

**Affiliations:** 1 Department of Cell and Developmental Biology, Rutgers University, New Brunswick, NJ 08901-8554, USA; joshuathomas2002@gmail.com; 2 Department of Biotechnology, American University of Ras Al Khaimah, Ras Al Khaimah P.O. Box 10021, United Arab Emirates; mohammedayyub799@gmail.com; 3 Adjunct Faculty, Department of Clinical Sciences, College of Dentistry, Ajman University, Ajman P.O. Box 346, United Arab Emirates; lovelyannamma@gmail.com; 4 Adjunct Faculty, Dental Research Cell, D. Y. Patil Dental College & Hospital, D. Y. Patil Vidyapeeth, Pune 411018, India; 5 Department of Basic Medical and Dental Sciences, College of Dentistry, Ajman University, Ajman P.O. Box 346, United Arab Emirates; 6 College of Dentistry, Al Ain University, Abu Dhabi P.O. Box 112612, United Arab Emirates; sabrin.azim@aau.ac.ae; 7 Department of Clinical Sciences, College of Dentistry, Ajman University, Ajman P.O. Box 346, United Arab Emirates; s.varma@ajman.ac.ae; 8 RAK College of Medical Sciences (RAKCOMS), RAK Medical & Health Sciences University (RAKMHSU), Ras Al Khaimah P.O. Box 11172, United Arab Emirates; 9 Department of Basic Medical and Dental Sciences, Ajman University, Ajman P.O. Box 346, United Arab Emirates

**Keywords:** epigenetics, gene therapy, CRISPR, RNA interference, epithelial carcinoma, genome instability, PRISMA

## Abstract

**Background**: Epithelial-derived carcinomas exhibit genomic instability due to genetic mutation and epigenetic dysregulation. Mechanisms such as DNA methylation, histone modifications, and noncoding RNA regulation may lead to tumor progression, immune evasion, and treatment resistance. Presently, gene-based and epigenetic therapies, including CRISPR systems, RNA interference, and chromatin-modifying agents, are reported to be effective in targeted cancer treatment. **Objectives**: This scoping review aims to map the evidence on gene-based technologies in epithelial-derived carcinomas, including therapeutic, diagnostic, prognostic, screening, and disease-modeling applications, and to identify molecular targets, translational outcomes, and gaps in the current evidence base. The review will characterize the molecular targets, gene-based approaches, cancer types, translational applications, and reported outcomes to identify evidence gaps, translational challenges, and priorities for future research. **Methods**: The scoping review was conducted as per PRISMA-ScR by conducting a literature search in databases such as PubMed, Embase, and Google Scholar, covering studies published between 2016 and 2026. In the inclusion criteria, all studies and reviews related to investigating gene-based, RNA-mediated, and epigenetic therapies in epithelial cancers were included. Thirty-six studies met the eligibility criteria. **Results**: Amongst the studies reviewed, the most common epithelial cancers reported were hepatocellular carcinoma, ovarian carcinoma, lung carcinoma, and pancreatic ductal adenocarcinoma. Among the epithelial-derived carcinomas identified in this review, HCC was the most frequently represented cancer; epigenetic alterations and therapies used were RNA-based therapies, CRISPR diagnostics, and epigenetic drugs. Ovarian and breast cancers targeting BRCA pathways and chromatin remodeling produced clinical translation outcomes. Lung and colorectal cancers showed moderate epigenetic involvement, often managed through combined therapeutic strategies. Pancreatic cancer therapies were limited by challenges in drug delivery despite identifiable molecular targets. Gene therapies such as CRISPR-based gene editing, RNAi/siRNA therapies, miRNA/lncRNA targeting, and HDAC inhibition were commonly reported. **Conclusions**: The present study indicates that the majority of epithelial-derived carcinomas can benefit from gene-based and epigenetic therapies. Though the majority of carcinomas, such as hepatocellular, have good clinical outcomes, pancreatic cancer has limited clinical translation.

## 1. Introduction

**Figure 1:**
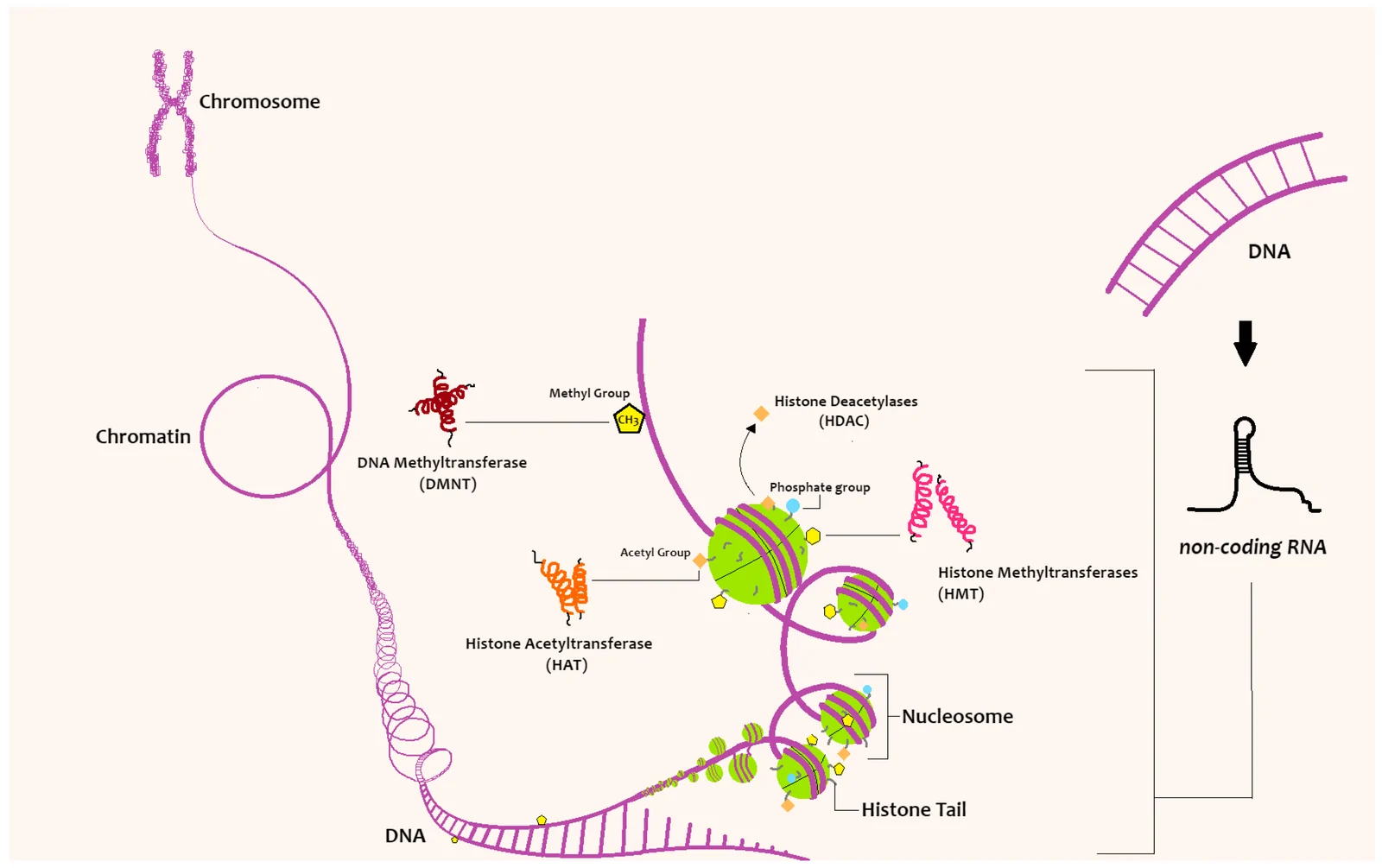
An overview of epigenetic modifications. This figure illustrates the various types of epigenetic modifications that regulate gene expression.

Epithelial-derived carcinomas are one of the most common reasons for cancer mortality [1]. Common epithelial-derived cancers include those of the breast, lung, colon, prostate, liver, ovary, and oral cavity [2]. In these cancers, epigenetic changes such as DNA methylation and histone modification often silence tumor-suppressor genes or activate pathways that support tumor growth [3], colorectal [4] and breast cancers that show promoter hypermethylation [5], while liver [6] and lung cancers [7] are influenced by altered noncoding RNAs and chromatin remodeling. These tumors arise from genetic alterations and disruptions in epigenetic regulation, causing genomic instability [8]. Epigenetic mechanisms are active drivers associated with tumor initiation, progression and drug resistance. Genomic integrity and DNA repair are maintained by mechanisms such as DNA methylation, histone modification, chromatin remodeling and noncoding RNA regulation [9]. Figure 1 shows that DNA methylation contributes to tumor development mainly by silencing tumor-suppressor genes when CpG islands in promoter regions become abnormally methylated [10]. This attracts proteins that condense chromatin, making genes inaccessible and permanently switched off. At the same time, global hypomethylation destabilizes the genome by activating transposable elements and increasing mutation rates. Together, these changes remove growth control and promote uncontrolled cell proliferation [11].

The type of histone modification influences the tumor development by altering chromatin structure, which in turn affects gene expression [12]. Markers such as H3K9, H3K27, and H4K20 cause methylation and thereby condense chromatin-silencing tumor-suppressor genes, disrupting normal transcriptional control [13]. Dysregulation of epigenetic enzymes, including methyltransferases and acetyltransferases, can lead to cellular proliferation and survival, while defects in histone variants such as CenH3 can impair chromosome segregation, contributing to aneuploidy and genomic instability [14,15]. Histone phosphorylation, when causing disruption of normal chromatin function at inappropriate sites or levels, leads to tumor proliferation [16]. For example, abnormal phosphorylation of specific residues in variants such as CenH3 can lead to aneuploidy [17]. In contrast, phosphorylation at serine 6 may not have any functional impact, highlighting the site-specific nature of this modification [18]. The combination of defects in histone variants like H3.3 and impaired support from noncoding RNAs causes genomic instability and tumor progression [19,20]. Short ncRNAs binding to complementary sequences may silence key tumor-suppressor genes, while long ncRNAs act as scaffolds, causing chromatin-modifying complexes, thereby altering gene expression [21]. These changes can promote epigenetic silencing, affect DNA repair processes, and create persistent regulatory loops favoring tumor growth [8].

Telomerase activation inhibits telomere shortening, disrupting normal control of chromosome stability contributing to tumor development [22,23]. Epigenetic therapy can reverse abnormal genes and suppress tumor progression by restoring normal cellular control [24]. Drugs such as HDAC inhibitors and DNA methylation modulators can reopen chromatin and increase the cancer cell’s sensitivity to treatment [25]. Gene therapies such as RNA-based and CRISPR systems have the potential to deliver to target tissues, provided efficient, tissue-specific delivery is achieved, which remains a major translational challenge. The challenges include biodistribution, stability, and off-target effects. If delivered efficiently, they can control cancer progression [26].

Gene-based and epigenetic therapies are used to diagnose and treat epithelial-derived carcinomas, but evidence remains heterogeneous across cancer types and treatment modalities. This scoping review aims to map the current evidence on gene-based technologies in epithelial-derived carcinomas, encompassing therapeutic, diagnostic, prognostic, screening, and disease-modeling applications. The review will characterize the molecular targets, gene-based approaches, cancer types, translational applications, and reported outcomes to identify evidence gaps, translational challenges, and priorities for future research.

## 2. Methods

### 2.1. Study Design

The search string included: (“Carcinoma” OR “Neoplasms” OR carcinoma* OR cancer* OR neoplasm* OR adenocarcinoma* OR “epithelial cancer*” OR “epithelial-derived cancer*” OR “squamous cell carcinoma*”) AND (“Gene Therapy” OR “gene therapy” OR “gene-based therapy” OR “gene-based technology” OR “gene editing” OR “genome editing” OR CRISPR OR “CRISPR-Cas9” OR “CRISPR-Cas12a” OR RNAi OR “RNA interference” OR siRNA OR miRNA OR microRNA* OR lncRNA* OR “antisense oligonucleotide*” OR “gene silencing” OR “gene targeting” OR “gene replacement” OR “genetic screening”) AND (epigenetic* OR “DNA methylation” OR “histone modification*” OR “histone acetylation” OR “histone methylation” OR DNMT* OR HDAC* OR “chromatin remodeling”) AND (therapy* OR treatment OR therapeutic* OR diagnosis* OR prognosis* OR biomarker* OR screening OR “disease model*” OR translational). The Embase strategy was adapted using corresponding Emtree terms and free-text keywords, with the same conceptual domains and publication period. Google Scholar searches used predefined term combinations covering gene therapy and gene-based technologies, RNA-based approaches, CRISPR/genome editing, epigenetic approaches, and diagnostic or prognostic applications in carcinoma and cancer. All searches were conducted on 31 March 2026, with the publication date restricted to March 2016 through March 2026.

**Figure 2:**
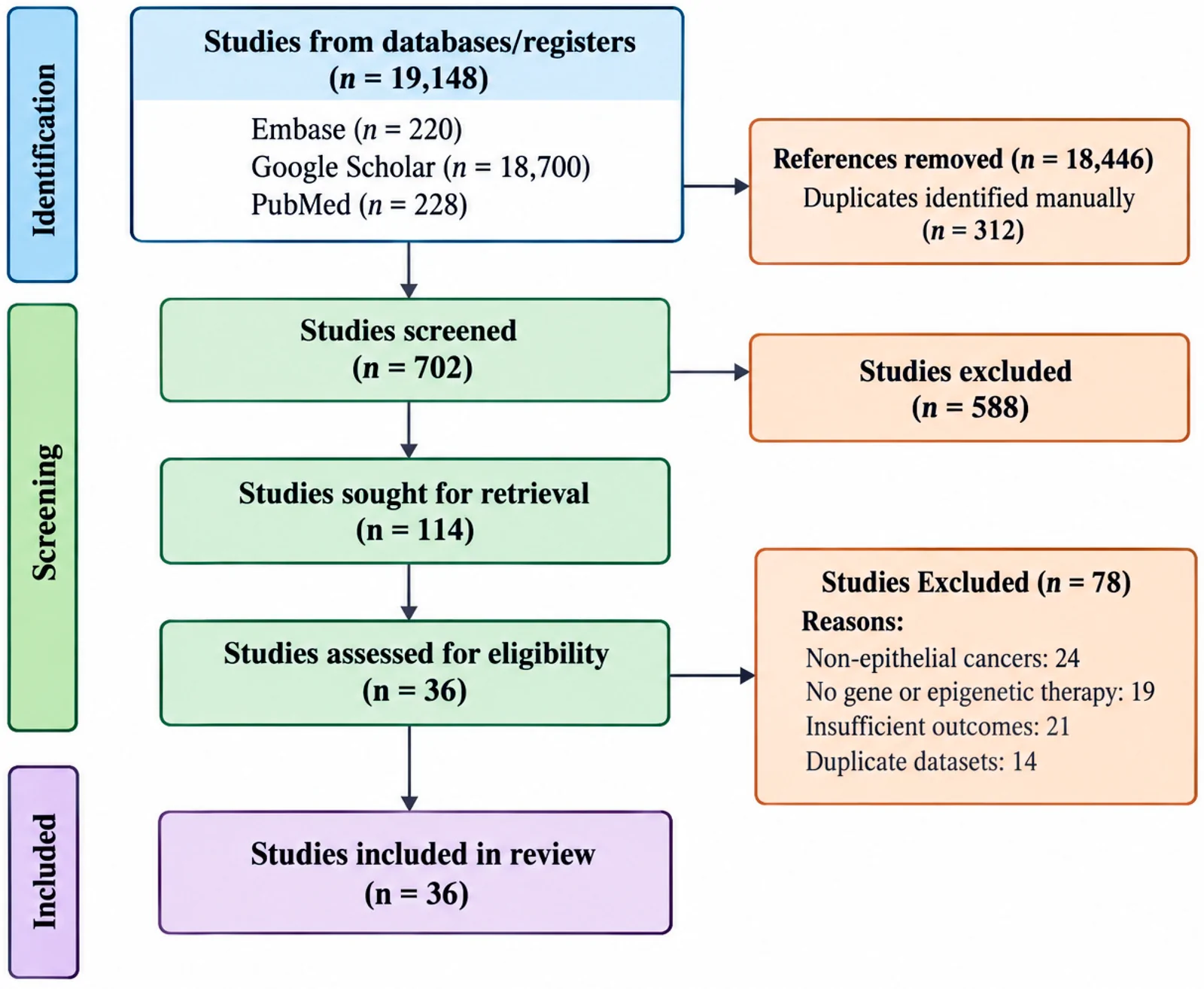
PRISMA flowchart.

The data identification and screening details are highlighted in the PRISMA flowchart, Figure 2. The electronic database and register searches initially identified 19,148 records. Following the removal of 18,446 records, including duplicates and records removed during the identification process, 702 records remained for title and abstract screening. The screening and eligibility assessment were subsequently conducted according to the PRISMA-ScR framework, with the reasons for exclusion documented at each stage. Three reviewers independently screened studies by assessing the titles and abstracts of all records retrieved. The same three reviewers independently performed full-text screening of retrieved articles based on inclusion and exclusion criteria. When eligibility remained unclear, the reviewers discussed the record and reached consensus with the other authors before making the final decision. The PRISMA flow chart cites reasons for exclusion based on the eligibility criteria. The selected studies were examined to confirm their relevance and outcomes. This scoping review was conducted in accordance with the Preferred Reporting Items for Systematic Reviews and Meta-Analyses Extension for Scoping Reviews (PRISMA-ScR) guidelines. The study followed identification, screening, eligibility assessment and inclusion, with evidence detailed in Figure 2. The completed PRISMA-ScR checklist is included in the Supplementary File, and study selection is presented in the PRISMA flow diagram.

### 2.2. Definition and Eligibility Criteria

Gene therapy was defined as the therapeutic modification, replacement, addition, or targeted alteration of genetic material to change disease biology or produce a therapeutic effect. This included target identification, functional screening or disease modeling.

RNA therapeutics were defined as approaches in which RNA molecules or RNA-mediated mechanisms are used to achieve a therapeutic effect, including siRNA, RNA interference, antisense oligonucleotides, therapeutic miRNAs, and related RNA-based gene-silencing strategies. This included biomarkers, diagnostic, and prognostic studies.

Epigenetic therapy was defined as an intervention that directly targets or modifies epigenetic processes, including DNA methylation, histone modification, chromatin remodeling, and related regulatory mechanisms.

CRISPR diagnostics were defined as diagnostic methods that use CRISPR-associated systems, including Cas proteins, to detect or quantify cancer-related nucleic acids or molecular biomarkers. Biomarkers included measurable molecular features used for cancer detection, diagnosis, prognosis, prediction of treatment response, treatment resistance or patient stratification. Studies were eligible when they investigated a gene-based, RNA-based, epigenetic, CRISPR-based, biomarker, or molecularly targeted approach relevant to cancer diagnosis, prognosis, treatment, treatment response, resistance, patient stratification, theranostics, or therapeutic development.

The inclusion criteria were peer-reviewed articles published in English. Eligible studies included original research, clinical trials, experimental studies, systematic reviews, meta-analyses, and narrative reviews that reported clinical, translational, or mechanistic evidence related to gene-based therapies in epithelial-derived carcinomas. Editorials, conference abstracts, commentaries, letters, protocols, and studies without relevant therapeutic or translational outcomes were excluded. The narrative reviews, systematic reviews, meta-analyses, diagnostic biomarker studies, CRISPR screening studies, and disease-modeling studies were included to capture the broader evidence in relation to the development and translation of gene-based therapies for epithelial-derived carcinomas. The systematic reviews and meta-analyses were included to identify established molecular targets, therapeutic strategies, and evidence trends. The diagnostic studies were included when they informed target identification, patient stratification, or treatment selection. CRISPR screening studies were included to identify potential therapeutic targets and genetic dependencies, and disease-modeling studies were included where they evaluated gene-based therapeutic mechanisms or models relevant to therapeutic development. This approach was adopted to identify evidence gaps across target discovery, preclinical development, and clinical translation. This scoping review was not registered.

### 2.3. Data Analysis

The data extracted include study type, epithelial cancer type, epigenetic mechanisms and genomic changes in the epithelial cancer. The extracted data also analyses the types of therapeutic interventions for epithelial cancers that are based on gene therapy or epigenetic mechanisms.

### 2.4. Data Synthesis

A descriptive analytical approach was used to categorize the findings involving the epigenetic mechanisms, pathways leading to genomic instability and gene therapies involved in each type of epithelial cancer.

## 3. Results

A total of 36 studies were included, comprising 26 reviews (72.2%), five meta-analyses (13.9%), three Phase I studies (8.3%), one Phase I/II study (2.8%), and one experimental study (2.8%) Table 1. The most frequently studied cancers were hepatocellular carcinoma, ovarian carcinoma, lung carcinoma, and pancreatic ductal adenocarcinoma. Across these studies, several therapeutic approaches were identified. Epigenetic therapies, particularly HDAC inhibitors, were commonly investigated in cancers such as HCC, breast, and renal cell carcinoma, where they were shown to influence tumor growth and immune evasion, supporting their use in combination with immunotherapy. RNA-based therapies have been shown to enhance drug delivery, indicating effective tumor treatment. Gene editing, such as CRISPR systems, identifies resistance pathways and enables early diagnosis through biomarker development and precision treatment. Combination therapies have been reported to show better clinical outcomes.

Analysis of the included studies categorizes the epigenetic and gene-based cancer therapies into epigenetic modulation, RNA-based therapies, CRISPR-mediated and combination strategies. In epigenetic approaches, HDAC inhibitors are linked to tumor suppression and immunomodulation in the treatment of hepatocellular, breast and renal cancers. The common types of gene mutation and epigenetic and gene therapies for each type of cancer are highlighted in Table 2.

The review included 36 studies. Hepatocellular carcinoma was the most frequently represented malignancy (11/36, 30.6%), followed by epithelial ovarian carcinoma and clear-cell renal cell carcinoma (4/36, 11.1% each), pancreatic ductal adenocarcinoma and lung carcinoma (3/36, 8.3% each), and head and neck squamous cell carcinoma and thyroid carcinoma (2/36, 5.6% each). Oral squamous cell carcinoma, breast carcinoma, colorectal carcinoma, adenocarcinoma, and squamous cell carcinoma were each represented by one study (1/36, 2.8%). The included evidence comprised 26 reviews (72.2%), five meta-analyses (13.9%), three Phase I studies (8.3%), one Phase I/II study (2.8%), and one experimental study (2.8%). Across the included studies, RNA-based approaches were the most frequently reported modality (18/36, 50.0%), encompassing RNA interference, miRNA, lncRNA, and related approaches addressing oncogenic signaling, angiogenesis, chemoresistance, and biomarker development. CRISPR-based technologies were reported in 11 studies (30.6%) and included genetic screening, molecular diagnostics, disease modeling, and therapeutic gene editing. Epigenetic modulation was reported in six studies (16.7%), primarily involving HDAC inhibition, epigenetic regulation, and RNA-mediated mechanisms associated with tumor suppression, immune modulation, and therapeutic resistance. Multimodal approaches were identified in five studies (13.9%), combining gene-based strategies with nanotechnology, immunotherapy, antiviral therapy, or targeted drug treatment. These categories overlap, as individual studies can incorporate more than one therapeutic modality, molecular target, mechanism, or translational application. The qualitative review of the 36 included studies demonstrates progress in gene-based therapies for epithelial-derived cancer. Biomarkers and genome-editing-based epigenetic modulation enable precision therapy for individual cancer patients. The following findings based on the qualitative data are:

1. Epigenetic modulation with histone deacetylase inhibition in epithelial-derived carcinoma such as hepatocellular carcinoma, breast cancer, and renal cell carcinoma suppressed tumor growth and reversed therapeutic resistance compared to monotherapy when combined with immune checkpoint inhibitors.
2. RNA-based therapeutics such as studies evaluating siRNA, miRNA, lncRNA, RNA interference (RNAi), and antisense oligonucleotides consistently targeted oncogenic signaling pathways, angiogenesis, chemoresistance, and metastatic progression across hepatocellular, ovarian, lung, pancreatic, thyroid, and squamous cell carcinomas. Circulating miRNA has been proved to be a potential diagnostic and prognostic biomarker.
3. CRISPR technologies such as CRISPR/Cas9, Cas12a, and CRISPR screening platforms were widely used to identify genetic dependencies, characterize mechanisms of drug resistance, detect circulating tumor DNA, and develop disease models for precision oncology.

The qualitative analysis of the data revealed several gaps, including that most of the studies were narrative reviews. The lack of large experimental studies and clinical trials providing direct clinical evidence was noted. The most reported clinical studies were on hepatocellular carcinoma, while the other epithelial carcinomas were underrepresented. The review evidence suggests that gene-based therapies lead to clinical translation, but long-term randomized studies and clinical trials are required to clinically evaluate their outcome.

## 4. Discussion

The scoping review indicates a rapidly expanding field of gene-based and epigenetic therapies for epithelial-derived carcinomas. Current approaches include RNA interference, noncoding RNA modulation, epigenetic therapy, CRISPR-based technologies, and tumor-directed nanomedicine. Major barriers include tumor heterogeneity, treatment resistance, delivery to malignant tissue, off-target effects, and the difficulty of translating molecular target engagement into meaningful clinical benefit.

The most often reported carcinoma, according to our research, was hepatocellular carcinoma (HCC). HCC shows global hypomethylation leading to chromosomal instability, and hypermethylation contributes to the silencing of tumor-suppressor genes such as *p16* and *RASSF1A* [73]. Other mechanisms such as overexpression of HAD Cs [74] and ncRNA dysregulation [75] promote tumor cells. The therapeutic advances for HCC are the reverse transcriptional repression of HDAC inhibitors combined with immunotherapy [76], siRNA targeting VEGF, suppressing angiogenesis [77] and CRISPR-based antiviral editing of HBV/HCV genomes [36]. Among the considered epigenetic therapies, histone deacetylase (HDAC) inhibitors represent the most clinically mature class, with clinical translation in hematologic malignancies and in solid tumors [78]. However, their application to epithelial cancers remains limited due to tumor selectivity, systemic toxicity, and the complexity of epigenetic regulation.

In ovarian cancer, the epigenetic DNA repair pathways are silenced, leading to hypermethylation of *BRCA1*, causing a BRCAness phenotype [79]. Histone modifications cause miRNA dysregulation [80]. Therapeutically, CRISPR-based editing is used experimentally to restore or model *TP53* and *BRCA* function and delivery systems for precision treatment response [81,82]. In breast cancer, the epigenetic alterations include DNA methylation [83] and histone deacetylation and transcriptional repression [84]. In inRNA dysregulation, miR-155 and miR-34 influence proliferation, metastasis and therapy resistance [85]. HDAC inhibitors represent the most clinically advanced epigenetic therapy in breast cancer, particularly in combination with endocrine therapy or chemotherapy [86,87].

**Figure 3:**
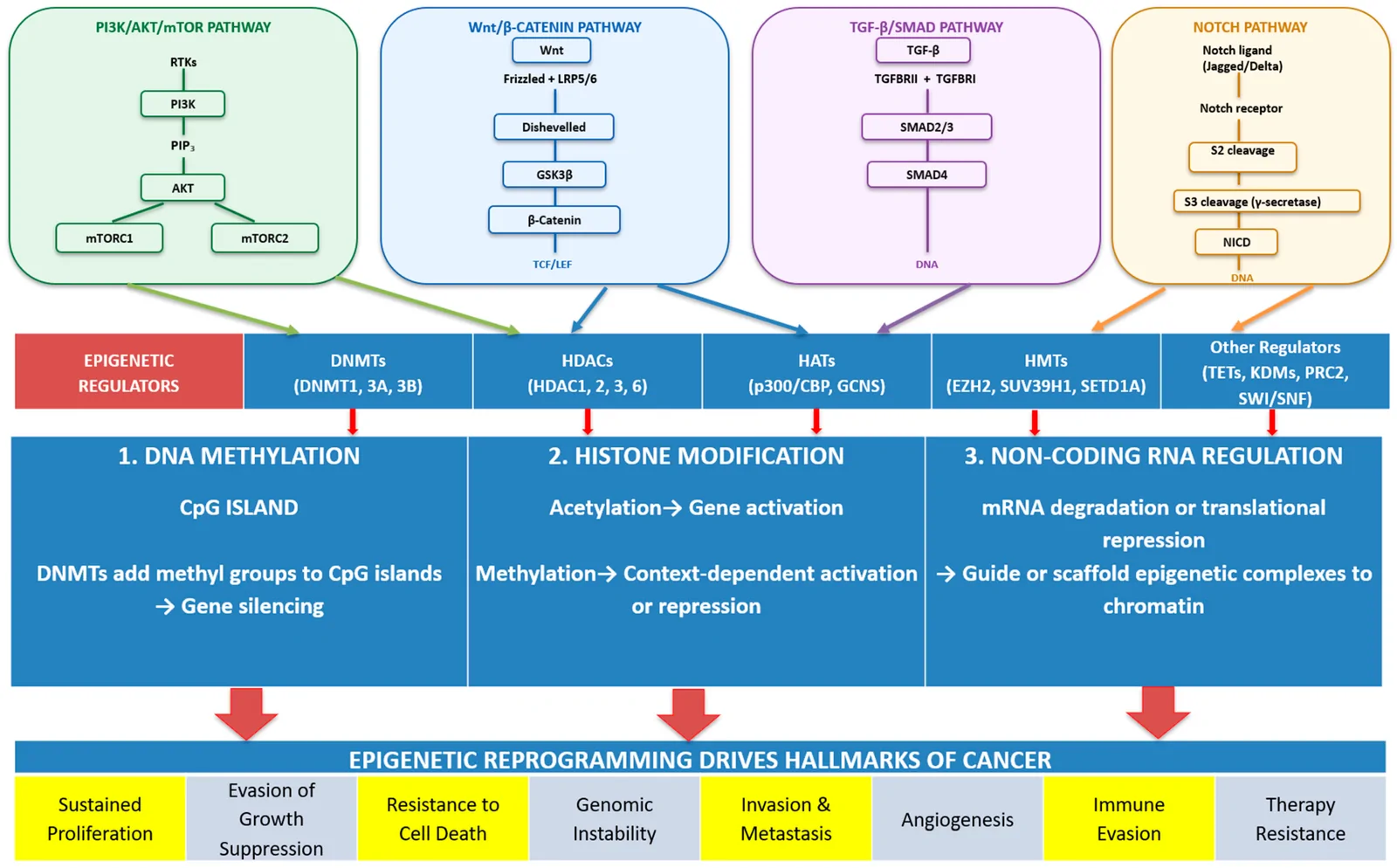
Epigenetic signaling pathways in ectodermal cancers.

In lung cancer, DNA methylation silences tumor-suppressor genes such as *CDKN2A* [88] and histone modifications promote cell proliferation and metastasis [89]. Therapies combine epigenetic modulation with targeted therapies such as demethylating agents and HDAC inhibitors along with immunotherapy [90]. Biomarkers such as UHRF1 have been proposed to guide treatment selection [91]. Recent therapeutic advances include targeting immune checkpoints such as TIGIT, suggesting that epigenetic regulation influences immune responsiveness [92]. Figure 3 shows a diagrammatic representation of epigenetic signaling pathways in ectodermal cancers. Nanoparticle-based delivery systems are under investigation to overcome this barrier [93]. In clear cell renal cell carcinoma (ccRCC), the epigenetic regulation of hypoxia pathways are influenced by DNA methylation and histone modifications. They affect genes involved in oxygen sensing and angiogenesis through HIF signaling [94]. Therapies include the use of siRNA targeting HIF-2α, tumor uptake and gene silencing in clinical trials [32,95]. HDAC inhibitors are also being explored to overcome resistance to targeted therapies [96]. In regard to the head and neck squamous cell carcinoma (HNSCC), diagnostic profiling [97] and RNA interference and CRISPR approaches targeting HPV oncogenes (E6/E7) are under investigation [98]. In the diagnostic aspect, miRNA-based diagnostics with sensitivity and specificity have been tried clinically [99]. In thyroid cancer, epigenetic DNA methylation patterns and miRNA profiles are used for tumor classification and prognostication rather than direct therapeutic targeting [100]. Other therapies include RNAi targeting survivin and experimental CRISPR delivery systems [101]. In pancreatic cancer, drug delivery remains a major barrier as tumor cells are within the dense, poorly vascularized pancreatic tumor microenvironment [102]. RNA-based therapies, such as siRNA and antisense approaches, due to chemical stabilization and nanoparticle or ligand-mediated delivery, have improved target specificity and intracellular uptake [103]. Nevertheless, in solid epithelial tumors, the approaches remain limited by factors such as tumor penetration, endosomal escape, and heterogeneous target expression. miRNA-based therapies are at an earlier stage, with preclinical evidence and limited clinical validation, partly because individual miRNAs can regulate multiple transcripts and may therefore produce unpredictable biological effects. Gene-delivery systems, including viral vectors, nanoparticles, and extracellular-vesicle-based platforms, are at an intermediate translational stage [101]. CRISPR-based genome editing has shown considerable potential for functional genomic screening and mechanistic validation; however, therapeutic genome editing faces additional challenges involving delivery, editing efficiency, tissue specificity, immune responses, and unintended genomic alterations [104]. Presently, the modality is in preclinical or early-phase studies and cannot be considered evidence of clinical effectiveness.

The scoping review demonstrates that the usefulness of these approaches depends on tumor biology. Amongst the epithelial-derived cancers, hepatocellular carcinoma, with its molecular heterogeneity and alterations involving TERT, TP53, CTNNB1, AXIN1, and chromatin-remodeling genes, indicates treatment approaches combining RNA-based and epigenetic treatments. Breast, lung, and colorectal carcinomas already have established molecularly targeted treatments to overcome resistance, improve treatment sensitivity, and refine patient selection. Pancreatic ductal adenocarcinoma shows challenges because of its complex genomic profile and restrictive tumor microenvironment. Ovarian carcinoma, head and neck squamous cell carcinoma, thyroid carcinoma, and cutaneous squamous cell carcinoma present opportunities to integrate RNA, epigenetic, and CRISPR-based approaches with established targeted or immune therapies.

### 4.1. Limitations

A total of 36 studies were included, comprising 26 reviews (72.2%), five meta-analyses (13.9%), three Phase I studies (8.3%), one Phase I/II study (2.8%), and one experimental study (2.8%). The distribution indicates a limitation in high-level clinical evidence, as the studies are in the translation phase.

The variability in cancer types and epigenetic modifications is vast, which cannot be covered in a scoping review. Among epithelial cancers, the most represented was hepatocellular carcinoma. Breast and colorectal carcinomas are less well covered, which may bias reporting.

Amongst the therapies, RNA-based and CRISPR technologies do not have long-term data compared to drug therapies. The challenges, such as delivery efficiency, tumor specificity, off-target effects, and long-term safety, do not have enough adequate data published.

Many review articles may overlap in the frequently reviewed cancer types and therapeutic strategies. Therefore, the findings should be interpreted as a map of the existing evidence rather than as independent estimates of the strength of therapeutic effects.

Finally, inter-patient variability and tumor heterogeneity, along with consistent biomarkers, limit the clinical predictability of treatment response.

### 4.2. Future Implications

The findings from this review suggest precise targeting of delivery systems such as nanoparticle carriers and extracellular vesicle platforms to improve safety and efficiency in RNA-based and gene-editing therapies. Precise biomarkers and epigenetic profiling will allow for better patient stratification and targeted therapeutic interventions. Identifying specific biomarkers such as miRNAs, methylation signatures, or chromatin states ensures efficient clinical outcomes.

## 5. Conclusions

The scoping review demonstrates that epigenetic alterations can cause progression in epithelial-derived cancers. The present treatment modalities include gene-based and epigenetic therapies. These therapies target oncogenic pathways, restoring tumor suppressor function and improving immune response.

Epigenetic and RNA-based therapies show considerable therapeutic potential across carcinomas, including HCC, but current evidence remains predominantly preclinical or derived from early-phase clinical studies. Although early trials have demonstrated feasibility and occasional disease stabilization, consistent improvements in objective response and progression-free survival have not yet been established. Further well-designed clinical trials are therefore required to determine whether the promising preclinical findings can translate into durable clinical benefit. The challenges, such as inefficient delivery, off-target effects, and the lack of robust long-term clinical data, remain.

The reviewed studies suggest that therapeutic combinations are tailored to tumor biology, overcoming drug resistance, and improving target specificity. The heterogeneity in evidence requires large disease-specific clinical trials.

## Figures and Tables

**Table 1:** Diagnostic, therapeutic and prognostic gene-based/epigentic/molecular clinical translation in epithelial-derived carcinomas.

No.	Study (First Author, Year, PMID)	Cancer Type (Epithelial)	Study Design	Gene-Based Modality	Primary Application	Translational Role	Primary Molecular Target	Reason for Inclusion
1	Sadia 2025, PMID: 40940750 [27]	Hepatocellular carcinoma	Review	Epigenetic modulation—HDAC inhibition	Therapeutic	Therapeutic development/combination therapy	Class I/II HDACs	The review addresses HDACs as therapeutic targets.
2	Lu 2025, PMID: 40651547 [28]	Pancreatic ductal adenocarcinoma	Review	CRISPR/genome editing—CRISPR/Cas9 screening	Target discovery/genetic screening	Target identification and validation	KRAS; immune modulators	CRISPR is used as a screening diagnostic tool.
3	Cen 2025, PMID: 40209498 [29]	Hepatocellular carcinoma	Review	CRISPR-based technology—CRISPR/Cas12a	Diagnostic	Diagnostic development	ctDNA/RNA biomarkers	The technology is used for detection of ctDNA/RNA diagnostic biomarkers.
4	Li 2025, PMID: 40054045 [30]	Epithelial ovarian carcinoma	Review	CRISPR/genome editing	Disease modeling	Disease modeling/therapeutic development	TP53; BRCA pathways	CRISPR genome editing in ovarian cancer therapeutic development.
5	Nair 2024, PMID: 39561094 [31]	Pancreatic ductal adenocarcinoma	Review	RNA-based technology—RNAi nanomedicine	Therapeutic	Delivery-enabled therapy	Notch; Hedgehog pathways	Directly concerns RNAi-based nanomedicine as a treatment strategy.
6	Alzahrani 2024, PMID: 38987439 [32]	Hepatocellular carcinoma	Review	RNA-based technology—siRNA	Therapeutic	Preclinical-to-clinical translation	VEGF; proliferation-associated genes	siRNA is discussed as a cancer-treatment modality, with progression from preclinical research to clinical translation.
7	Easwaran 2025, PMID: 38808710 [33]	Oral squamous cell carcinoma	Review	CRISPR + phage delivery/theranostic technology	Theranostic	Targeted delivery and integrated diagnosis/therapy	Integrin αvβ6	Bacteriophage-based theranostics—diagnostic and therapy.
8	Brugarolas 2024, PMID: 38652038 [34]	Clear–cell renal cell carcinoma	Phase I	RNA-based technology—siRNA	Therapeutic	First-in-human clinical translation	HIF-2α	First-in-human clinical evaluation of an siRNA therapeutic targeting HIF-2α.
9	Amjad 2024, PMID: 38361756 [35]	Hepatocellular carcinoma	Review	CRISPR/genome editing	Target discovery/therapeutic development	Resistance mechanism identification	Resistance-associated genes	CRISPR genome editing in HCC—therapeutic preclinical development.
10	Bartosh 2023, PMID: 38203503 [36]	Virus-associated HCC	Review	CRISPR/genome editing	Therapeutic/preventive	Antiviral cancer prevention	HBV/HCV genomes	CRISPR targets HBV/HCV genomes. It is indirectly based on association with HCC, rather than tumor itself.
11	Godbole 2023, PMID: 37820858 [37]	Epithelial ovarian carcinoma	Review	CRISPR/genome editing + extracellular-vesicle delivery	Therapeutic	Delivery and safety optimization	Oncogenic loci	EV-mediated delivery of CRISPR systems for cancer therapy.
12	Coelho 2022, PMID: 36307400 [38]	Epithelial ovarian carcinoma	Experimental	CRISPR screening	Target discovery/patient stratification	Predictive biomarker development	BRCA2; ATM	CRISPR-Cas9 is used as a functional screening tool to identify genetic dependencies and predictive biomarkers. Diagnostics.
13	Wang 2022, PMID: 35834017 [39]	Adenocarcinomas	Review	RNA-based technology—CRISPRi/RNAi	Therapeutic	Gene repression/RNA therapeutics	ncRNAs; oncogenes	Addresses CRISPRi and RNAi as therapeutic approaches.
14	Kong 2021, PMID: 34665528 [40]	Hepatocellular carcinoma	Review	CRISPR + nanotheranostics	Theranostic	Integrated diagnosis and therapy	Tumor-driving genes	Combines CRISPR technology with nanotheranostics for integrated cancer diagnosis and treatment.
15	Wang 2021, PMID: 34163045 [41]	Hepatocellular carcinoma	Review	Functional genetic screening	Target discovery/genetic screening	Precision drug discovery	Tumorigenic pathways	CRISPR/genetic screening is a research tool for identifying tumorigenic pathways and drug targets.
16	Zhou 2021, PMID: 33845800 [42]	Head & neck SCC	Meta-analysis	RNA-based technology—miRNA	Diagnostic	Biomarker validation	miR-221-3p	The study evaluates miRNA as a biomarker. miR-221-3p expression.
17	Fathi 2021, PMID: 33686787 [43]	Colorectal carcinoma	Review	Gene-targeted immune modulation	Therapeutic/combination therapy	Immunotherapy optimization	TIGIT	TIGIT cancer immunotherapy target.
18	Gowhari Shabgah 2021, PMID: 33625758 [44]	Hepatocellular carcinoma	Review	Gene targeting	Therapeutic	Targeted therapy development	Midkine (MDK)	Midkine is discussed as a molecular target.
19	Wu 2020, PMID: 32487115 [45]	Hepatocellular carcinoma	Review	CRISPR/Cas9	Therapeutic	Targeted gene editing	Oncogenic drivers	CRISPR/Cas9 as a potential therapeutic approach to HCC.
20	Goto 2020, PMID: 32212938 [46]	Lung carcinoma	Meta-analysis	Epigenetic biomarker	Prognostic	Prognostic stratification	UHRF1	UHRF1 is evaluated as a prognostic/epigenetic biomarker.
21	Wood 2020, PMID: 31654285 [47]	Clear–cell RCC	Phase I	Epigenetic modulation + targeted therapy	Therapeutic	Combination clinical therapy	HDAC; mTOR	Therapeutic intervention combining epigenetic modulation with targeted therapy.
22	Wei 2019, PMID: 31651347 [48]	Hepatocellular carcinoma	Review	RNA-based technology—ncRNA targeting	Therapeutic	Resistance management	miRNAs; lncRNAs	ncRNAs in HCC drug resistance and their potential therapeutic exploitation.
23	Zucchetti 2019, PMID: 30553187 [49]	Breast carcinoma	Review	Epigenetic modulation—HDAC inhibition	Therapeutic	Translational therapy	Histone acetylation/HDACs	HDAC inhibition as an anticancer therapeutic strategy with translational relevance.
24	Adamska 2018, PMID: 30090003 [50]	Pancreatic ductal adenocarcinoma	Review	Gene-targeted molecular therapy	Prognostic/predictive	Chemoresistance and treatment stratification	ABC transporters	ABC transporters as prognostic predictions.
25	Xiong 2018, PMID: 30036873 [51]	Lung adenocarcinoma	Meta-analysis	RNA-based technology—lncRNA targeting	Therapeutic/prognostic	Target and biomarker development	NEAT1	NEAT1 expression and potential biomarker for diagnostics.
26	Santarelli 2018, PMID: 29587347 [52]	Squamous cell carcinoma	Review	RNA-based technology—RNAi/antisense	Therapeutic	Sensitization/combination therapy	Survivin (BIRC5)	RNAi/antisense approaches. Therapeutic sensitization.
27	Pratap 2018, PMID: 29516903 [53]	Lung carcinoma	Review	RNA-based technology—miRNA profiling/targeting	Diagnostic + therapeutic	Precision oncology	Oncogenic miRNAs	Oncogenic miRNAs and their potential therapeutic relevance.
28	Lanzafame 2018, PMID: 29495592 [54]	Hepatocellular carcinoma	Review	RNA-based technology—lncRNA targeting	Diagnostic/therapeutic	Biomarker and target development	Dysregulated lncRNAs	Dysregulated lncRNAs as potential biomarkers and therapeutic targets.
29	Liang 2018, PMID: 29207053 [55]	Hepatocellular carcinoma	Meta-analysis	RNA-based technology—miRNA biomarker	Diagnostic	Early detection/biomarker validation	miR-338-5p	miR-338-5p is evaluated as a biomarker for HCC detection.
30	Ding 2017, PMID: 28849100 [56]	Hepatocellular carcinoma	Meta-analysis	RNA-based technology—miRNA restoration	Therapeutic	Therapeutic restoration	miR-136-5p	miR-136-5p might have therapeutic potential.
31	Rodríguez-Rodero 2017, PMID: 28440770 [57]	Thyroid carcinoma	Review	Epigenetic modulation	Diagnostic/stratification	Molecular classification	DNA methylation; miRNAs	Molecular classification involving DNA methylation and miRNAs as diagnostic affiliation.
32	Shaikh 2017, PMID: 28438299 [58]	Head & neck SCC	Review	RNAi + CRISPR	Therapeutic	Targeted gene silencing/combination therapy	HPV; oncogenes	RNAi and CRISPR as therapeutic approaches for head and neck cancer.
33	Chou 2017, PMID: 28294980 [59]	Papillary thyroid carcinoma	Review	RNA-based technology—miRNA targeting	Prognostic	Recurrence prediction	miR-146b	miR-146b, a potential therapeutic target.
34	Retamales-Ortega 2017, PMID: 28245631 [60]	Epithelial ovarian carcinoma	Review	RNA-based technology—miRNA regulation	Therapeutic	Targeted therapy development	NGF/TRKA	miRNA regulation of NGF/therapeutic potential.
35	Pili 2017, PMID: 28222071 [61]	Clear–cell RCC	Phase I/II	Epigenetic modulation + targeted therapy	Therapeutic	Clinical combination therapy	HDAC; VEGF	Clinical combination treatment involving HDAC inhibition and VEGF-targeted therapy.
36	Aggarwal 2017, PMID: 28221861 [62]	Renal cell carcinoma	Phase I	Epigenetic modulation—HDAC inhibition	Therapeutic	Resistance-reversal strategy	HDAC2	Clinical Phase I evaluation of an HDAC inhibitor-based combination.

**Table 2:** Types of cancer, including gene mutations and types of gene targeted therapy and epigenetic modalities.

Cancer (Epithelial Origin)	Most Frequent/Clinically Important Mutations (Selected)	Gene-Based/Genetic Therapeutic Approaches	Epigenetic/Gene-Regulatory Therapeutic Approaches
Hepatocellular carcinoma (HCC)	TERT promoter increase activity, TP53, CTNNB1 (β-catenin), AXIN1, ARID1A	RNA interference (RNAi) and siRNA-based approaches; tumor-directed nucleic-acid delivery; experimental CRISPR/Cas-mediated gene editing or gene regulation; miRNA- and lncRNA-directed strategies	HDAC inhibitors; DNA-methylation modulators; targeting of aberrant chromatin and transcriptional regulation [63].
Breast carcinoma	PIK3CA, TP53, ERBB2 (HER2 amplification), BRCA1/2 (subset), ESR1 mutations (ER+)	siRNA and antisense oligonucleotide approaches; experimental CRISPR/Cas-mediated gene editing; miRNA-based gene regulation; experimental gene-silencing strategies	HDAC inhibitors; DNA-methylation modulation; chromatin-modifying strategies; miRNA-mediated epigenetic regulation [64].
Non-small cell lung carcinoma (NSCLC) (epithelial)	KRAS (incl. G12C), EGFR (L858R, exon19del), ALK fusions, ROS1, BRAF	RNA-based therapeutic approaches, including siRNA and antisense strategies; experimental CRISPR/Cas-mediated gene editing or gene regulation; targeted nucleic-acid delivery	DNA-methylation modulation; HDAC inhibition; histone/chromatin-modifying approaches; epigenetic strategies investigated for radiosensitisation and immunomodulation [65].
Colorectal carcinoma	APC; TP53; KRAS; NRAS; BRAF V600E; PIK3CA; mismatch-repair deficiency involving MLH1, MSH2, MSH6, PMS2; SMAD4	RNAi/siRNA and antisense approaches; miRNA replacement or inhibition; lncRNA-directed strategies; experimental CRISPR/Cas-mediated gene editing or gene regulation	DNA-methylation modulators; histone-modifying approaches; epigenetic reprogramming; miRNA/lncRNA-mediated regulation [66].
Pancreatic ductal adenocarcinoma (PDAC)	KRAS (~90%); TP53; CDKN2A; SMAD4; less frequent BRCA1/2, PALB2, ATM alterations	siRNA/RNAi targeting oncogenic drivers, particularly KRAS; nanoparticle- and extracellular-vesicle-mediated nucleic-acid delivery; experimental CRISPR/Cas-based gene editing and gene regulation	DNA-methylation modulation; HDAC inhibition; chromatin-modifying strategies; epigenetic approaches targeting tumor plasticity and treatment resistance [67].
Epithelial ovarian carcinoma	TP53 (particularly frequent in HGSOC); BRCA1/2; HRD-associated alterations; PIK3CA, KRAS, ARID1A in selected histologic subtypes	siRNA and antisense approaches; miRNA-based therapeutic strategies; extracellular-vesicle-mediated delivery of therapeutic nucleic acids; experimental CRISPR/Cas approaches	HDAC inhibition; DNA-methylation modulation; chromatin-remodeling strategies; EV-based delivery, CRISPR preclinical models, lncRNA/miRNA biomarker & target work [68].
Head & neck squamous cell carcinoma (HNSCC)	TP53; PIK3CA; NOTCH1; CDKN2A; FAT1; KMT2D; NSD1; HPV-positive tumors characterized by viral E6/E7 oncogenes	RNAi/siRNA approaches; antisense and miRNA-based strategies; experimental CRISPR/Cas-mediated gene editing or gene regulation	HDAC inhibitors; DNA-methylation modulators; RNAi approaches, CRISPR screens; epigenetic inhibitors (HDACi) as immunomodulators [69].
Thyroid carcinoma (papillary)	BRAF V600E; RAS mutations; RET fusions; NTRK fusions; TERT promoter alterations	Targeted kinase inhibitors for advanced disease (RET, BRAF/MEK)	DNA-methylation modulation; histone/chromatin modification; epigenetic regulation of aberrant thyroid cancer transcriptional programs; miRNA-based biomarker/therapeutic research; epigenetic signatures under study [70].
Clear cell renal cell carcinoma (ccRCC) (epithelial kidney tubule origin)	VHL loss/inactivation; PBRM1; SETD2; BAP1; KDM5C; alterations in the HIF pathway	Tumor-directed HIF2α siRNA, including ARO-HIF2; experimental nucleic-acid delivery; CRISPR/Cas-based gene regulation and gene-editing approaches	Epigenetic approaches targeting chromatin remodeling and histone-regulatory pathways associated with PBRM1, SETD2, BAP1, and KDM5C alterations. Tumor-directed siRNA (ARO-HIF2) early trials demonstrated target knockdown but limited responses; epigenetic interventions explored [71].
Cutaneous squamous cell carcinoma (cSCC)	TP53; NOTCH1/2; CDKN2A; TERT promoter alterations; FAT1; KMT2C/KMT2D; NFE2L2	RNAi/siRNA-mediated gene silencing; antisense approaches; miRNA-based strategies; experimental CRISPR/Cas-mediated gene editing or gene regulation [71]	Epigenetic and immunomodulatory strategies emerging (HDACi research, immune combinations) [72].

## Data Availability

No new data were created or analyzed in this study. Data sharing is not applicable to this article.

## Supplementary Materials

The following supporting information can be downloaded at: https://www.mdpi.com/article/10.3390/biomedicines14102234/s1, PRISMA checklist.

## Abbreviations

ABC transporters	ATP-binding cassette transporters
ADP	Adenosine diphosphate
ATM	Ataxia telangiectasia mutated
BIRC5	Baculoviral IAP repeat-containing protein 5 (Survivin)
BRCA	Breast cancer gene
Cas	CRISPR-associated protein
ccRCC	Clear cell renal cell carcinoma
CDKN2A	Cyclin-dependent kinase inhibitor 2A
CpG	Cytosine
phosphate	guanine dinucleotide
CRISPR	Clustered regularly interspaced short palindromic repeats
ctDNA	Circulating tumor DNA
cSCC	Cutaneous squamous cell carcinoma
DNA	Deoxyribonucleic acid
DNMT	DNA methyltransferase
DNMT inhibitors	DNA methylation modulators
EGFR	Epidermal growth factor receptor
EV	Extracellular vesicle
H3K4	Histone H3 lysine 4
H3K9	Histone H3 lysine 9
H3K27	Histone H3 lysine 27
H4K20	Histone H4 lysine 20
HBV	Hepatitis B virus
HCV	Hepatitis C virus
HCC	Hepatocellular carcinoma
HDAC	Histone deacetylase
HDAC inhibitors	Histone deacetylase inhibitors
HIF	Hypoxia-inducible factor
HNSCC	Head and neck squamous cell carcinoma
HRD	Homologous recombination deficiency
ICI	Immune checkpoint inhibitor
KMT	Lysine methyltransferase
KRAS	Kirsten rat sarcoma viral oncogene homolog
lncRNA	Long noncoding RNA
MDK	Midkine
miRNA	MicroRNA
mTOR	Mechanistic target of rapamycin
ncRNA	Noncoding RNA
NEAT1	Nuclear enriched abundant transcript 1
NGF	Nerve growth factor
NSCLC	Non-small cell lung carcinoma
PARP	Poly (ADP-ribose) polymerase
PDAC	Pancreatic ductal adenocarcinoma
PRMT	Protein arginine methyltransferase
RNA	Ribonucleic acid
RNAi	RNA interference
siRNA	Small interfering RNA
SMAD4	SMAD family member 4
TE	Transposable element
TIGIT	T cell immunoreceptor with Ig and ITIM domains
TME	Tumor microenvironment
TP53	Tumor protein p53
TRKA	Tropomyosin receptor kinase A
TPE	Telomere position effect
UHRF1	Ubiquitin-like with PHD and RING finger domains 1
VEGF	Vascular endothelial growth factor
